# Schizophrénie et usage des anti-inflammatoires: la piste des anti-inflammatoires

**DOI:** 10.1192/j.eurpsy.2023.2299

**Published:** 2023-07-19

**Authors:** R. Mama Soumeya

**Affiliations:** Etablissement hospitalier spécialisé en psychiatrie sidi chami, oran, Algeria

## Abstract

**Introduction:**

l’actualité de la recherche en psychiatrie se focalise sur la possibilité de nouvelles pistes de prise en charge notamment celles qui sont basées sur la chimiothérapie, et ce, en dépassant le spectre de la prescription des neuroleptiques.

L’actualité de la recherche en psychiatrie se focalise sur la possibilité de nouvelles pistes deprise en charge notamment celles qui sont basées sur la chimiothérapie, et ce, en dépassant lespectre de la prescription des neuroleptiques.

**Objectives:**

la place des anti-inflammatoires dans la prise en charge de la schizophrénie

**Methods:**

Une étude publiée dans l’American Journal of Psychiatry suggère qu’une plus grande activité microgliale peut être un signe de schizophrénie. Ainsi, la détecter et la réduire pourrait être un élément de prévention de cette maladie. Une étude publiée dans l’American Journal of Psychiatry suggère qu’une plus grandeactivité microgliale peut être un signe de schizophrénie. Ainsi, la détecter et la réduire pourraitêtre un élément de prévention de cette maladie.

**Results:**

Il a été constaté qu’une activité microgliale élevée coïncide avec la réduction du volume de la matière grise chez les sujets atteints ou à risque de développer la schizophrénie. Ce paramètre est aussi un signe de gravité symptomatique chez les patients atteints de cette maladie.

Il a été constaté qu’une activité microglialeélevée coïncide avec la réduction du volume de la matière grise chez les sujets atteints ou à risquede développer la schizophrénie. Ce paramètre est aussi un signe de gravité symptomatique chezles patients atteints de cette maladie.

**Conclusions:**

Cette étude ouvre une nouvelle voie dans le traitement de la schizophrénie, par l’ajout au traitement de molécules ayant des propriétés anti-inflammatoires chez les patients présentant une résistance au traitement antipsychotique.

**Disclosure of Interest:**

None Declared

